# A meta-analysis of the effects of transcranial magnetic stimulation on hand function and daily living ability after stroke

**DOI:** 10.1097/MD.0000000000044029

**Published:** 2025-08-29

**Authors:** Yue Shen, Jinchao Du, Xiaoduo Yao, Jiqin Tang

**Affiliations:** a School of Rehabilitation Medicine, Weifang Medical University, Weifang, Shandong Province, China; b School of Rehabilitation Medicine, Shandong University of Traditional Chinese Medicine, Jinan, Shandong Province, China.

**Keywords:** ability of daily living, hand function, meta-analysis, stroke, transcranial magnetic stimulation

## Abstract

**Background:**

To compare the effects of different schemes of repeated transcranial magnetic stimulation (rTMS) on hand function and activities of daily living in stroke patients through meta-analysis.

**Methods:**

Randomized controlled trials were retrieved from China National Knowledge Infrastructure (CNKI), Wanfang Database, Chinese Scientific Journal Database (VIP), PubMed, Embase and Cochrane Library databases for the time period from the time of construction to October 2023. Two researchers independently screened the articles and extracted data. Data analysis was performed using RevMan 5.3 and Stata 14.0 software.

**Results:**

A total of 29 studies with a total of 1294 patients were included, involving 5 measures including low frequency combined with high frequency rTMS (LF-HF rTMS), low frequency rTMS (LF-rTMS), high frequency rTMS (HF-rTMS), theta burst stimulation (TBS) and conventional therapy. Wolf motor function test (WMFT) results showed that the effect of LF-rTMS group was significantly higher than that of conventional group (*P* < .05), and the ranking was better than that of TBS group and HF-rTMS group. The grip strength results showed that the effect of each group was better than that of the conventional group, although the difference was not significant, and the LF-HF rTMS group was the best. The results of modified barthel index (MBI) and Fugl-Meyer assessment of upper extremity (FMA-UE) showed that the treatment effect of LF-HF rTMS group was significant (*P* < .05), and the effect was the best in each group.

**Conclusion:**

Transcranial magnetic stimulation has a good improvement effect, and the LF-rTMS group has a better effect in the treatment of hand function. The LF-HF rTMS regimen combined with 2 kinds of magnetic stimulation has the best ranking in improving grip strength, activities of daily living and upper limb function, which has great application potential.

## 1. Introduction

Stroke is one of the most common diseases endangering human health, and its incidence has been increasing in recent years with a trend of younger age, among which more than 65% of patients have upper limb motor dysfunction,^[1]^ and upper limb dysfunction, especially hand dysfunction, seriously affects the ability of patients to perform activities of daily living.^[2,3]^ However, the optimal treatment of poststroke dysfunction, especially hand dysfunction, is still controversial.

Repeated transcranial magnetic stimulation (rTMS) is a noninvasive brain stimulation technique, which is safe, painless, and convenient. It utilizes magnetic stimulation to noninvasively affect the corresponding motor areas of the brain, and is widely used in the treatment of poststroke dysfunction with good therapeutic effects.^[4]^ rTMS can mainly include low frequency rTMS (LF-rTMS), high frequency rTMS (HF-rTMS), theta burst stimulation (TBS) and low frequency combined with high frequency rTMS (LF-HF rTMS).^[5]^ During treatment, studies applying only one type of rTMS stimulation for treatment are more common, and it is less common for treatment protocols combining 2 types of rTMS to be applied, but the studies by Long et al^[6]^ and Chen et al,^[7]^ among others, have indicated that LF-HF rTMS also has a better therapeutic effect.

In terms of therapeutic principle, rTMS is designed to improve the balance between the cerebral hemispheres by increasing the excitability of the affected hemisphere with HF-rTMS and inhibiting the excitability of the healthy hemisphere with LF-rTMS, thus promoting the recovery of limb function. Currently, the optimal treatment protocol for hand dysfunction remains controversial and has not yet been investigated in a net meta-analysis. The uniqueness and contribution of this study is the inclusion of more protocols to compare the treatment effects between different protocols, and the aim of this study is to inform the choice of treatment protocols for rTMS through a meta-analysis of treatments for hand dysfunction.

## 2. Materials and methods

This study is registered with PROSPERO under the registration number CRD42023477179.

### 2.1. Search strategy

The strategy for identifying eligible randomized controlled trials involved a comprehensive search in China National Knowledge Infrastructure (CNKI), Wanfang Database, Chinese Scientific Journal Database (VIP), PubMed, Embase, and Cochrane Library databases. This was achieved by integrating subject terms with free-text words. The search formula employed in PubMed, presented as a representative example, is as follows: (Stoke[MeSH] OR CVA) AND (transcranial magnetic stimulation[MeSH] OR rTMS OR repetitive transcranial stimulation OR repetitive transcranial magnetic stimulate OR Theta OR noninvasive brain stimulation) AND (Hand[MeSH] OR upper limb OR hand or manual ability OR fine movement OR fine motion OR activities of daily living OR ADL).

### 2.2. Inclusion and exclusion criteria

#### 2.2.1. Inclusion criteria

Type of study: A randomized controlled trial of different protocols of transcranial magnetic stimulation to improve hand function and its related mobility after stroke. Study subjects: patients met the diagnostic criteria of stroke or were diagnosed by relevant imaging means; patients were > 18 years old and conscious; the sites of transcranial magnetic stimulation were all located in the motor area of the cerebral cortex; there was no restriction on gender and duration of the disease. Interventions: Comparison of the 4 treatment measures of LF-HF rTMS, LF-rTMS, HF-rTMS and TBS with sham stimulation or with conventional therapies, or intercomparison between the 4 rTMS modalities. Outcome indexes: Wolf motor function test (WMFT), Fugl-Meyer assessment of upper extremity (FMA-UE), Modified Barthel Index (MBI) and hand grip strength were used as outcome indicators. ①The WMFT is primarily used to assess hand motor function and consists of 15 task items for simple joint movements and compound functional movements, each of which is categorized into 6 levels. The scale has a total score of 75 points, and the higher the score, the better the motor function of the hand. ②hand grip strength is an indicator of muscle strength assessment, where the patient grasps the grip with maximum force with one hand, and the higher the value obtained from the measurement, the stronger the muscle strength on that side. ③The MBI is a tool used to assess a patient’s ability to perform activities of daily living with 11 items out of 100, with higher scores suggesting a better ability to perform activities of daily living. ④The FMA-UE is a clinically important assessment tool for measuring upper extremity motor function after stroke, with a total score of 66, with higher scores suggesting better upper extremity motor function.

#### 2.2.2. Exclusion criteria

Unclear provision of trial data and difficulty in accurately calculating data for outcome indicators. Inconsistency of interventions. Duplication of literature or inability to be accessed in its entirety. Low-quality studies or non-RCT types of studies. Animal-based experiments and reviews.

### 2.3. Literature screening and data extraction

Two researchers used EndNote X9 software to remove duplicates and then initially screened the titles and abstracts of the articles. Through a detailed examination of the complete texts, they ultimately selected 29 papers. The data extraction process was mainly handled by 2 researchers which involved gathering information such as first author, year of publication, sample size, stimulus location, and outcome metrics.

### 2.4. Quality evaluation of included studies

The risk of bias was assessed by 2 researchers according to the method recommended by the Cochrane Collaboration,^[8]^ and the risk of bias summary chart was drawn by the software Review Manager 5.3. During the evaluation process, the researchers completed the evaluation independently and then cross-checked, and if differences were encountered, they discussed and resolved the differences with the third researcher.

### 2.5. Statistical methods

The risk of bias summary graphs was plotted through the software Review Manager 5.3. Frequency meta-analysis and related graphs were mainly realized by Stata 14.0 software. Standardized mean difference and 95% CI were used as effect size indicators for continuous data. Inconsistency tests and reticulation plots were first performed. When the global inconsistency result test was performed, if the difference between direct and indirect comparisons was insignificant, *P* > .05. Local inconsistency was tested by the node splitting method, and the inconsistency test judged the closed-loop consistency of each outcome indicator. Secondly, the cumulative sorting probability graph was plotted by software and sorted according to the area value under the cumulative curve in the graph. The sorting was applied to judge the best stimulation method. The article compares the treatment effects of each intervention in detail through drawing league tables. Finally, the article examines publication bias through the drawing of funnel plots.

## 3. Results

### 3.1. Literature search results

**Figure 1.**
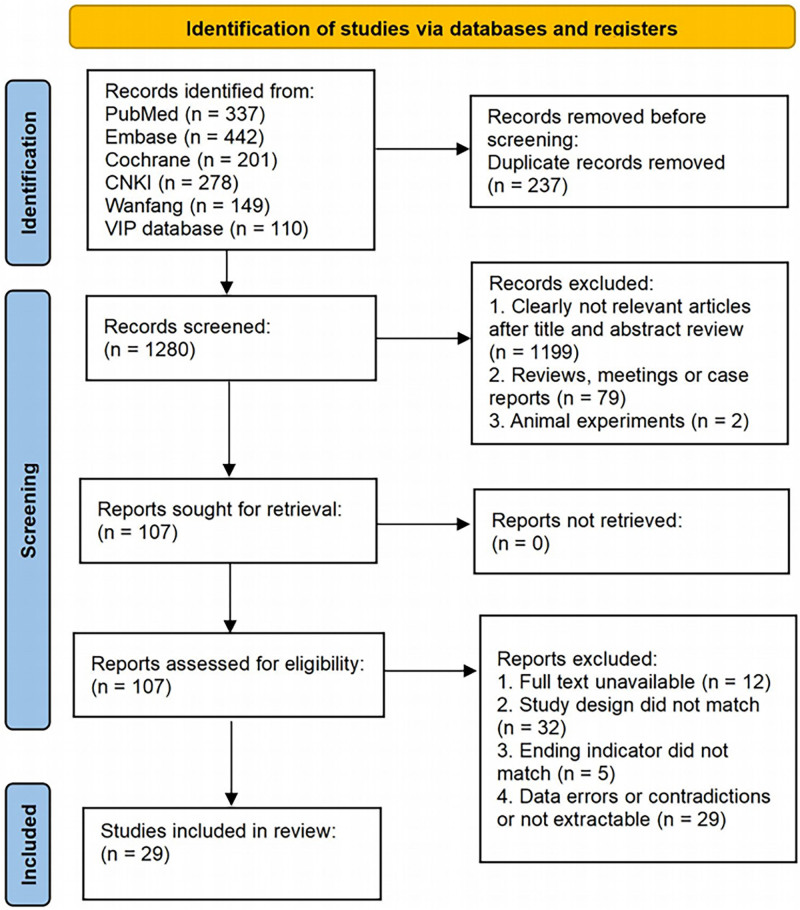
Flow diagram of literature screening and selection process.

A total of 1517 documents were obtained after searching the corresponding databases, and 29 documents were finally included after screening.^[9–37]^ The specific screening process is shown in Figure 1.

### 3.2. Basic characteristics and quality assessment of the included literature

**Figure 2.**
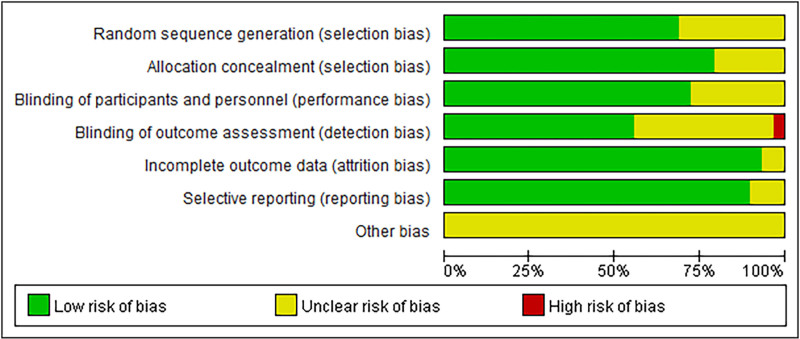
Risk of bias summary chart.

Among the 29 articles included in the study, there were 11 articles in Chinese and 18 articles in foreign languages, involving 22 2-arm studies, 6 3-arm studies and 1 4-arm study, with a total of 1294 patients. Among the 29 articles, 18 articles were related to LF-rTMS, 13 articles were related to HF-rTMS, 3 articles were related to LF-HF rTMS, and 6 articles were related to TBS. The 4 interventions and 1 control intervention were A = LF-HF rTMS, B = LF-rTMS, C = HF-rTMS, D = TBS, and E = Regular intervention, respectively. The basic characteristics of these studies are shown in Table 1, and the assessment of the risk of bias is summarized in Figure 2.

**Table 1:** The characteristic of included studies.

Study ID	Sample size (e/c)	Age (e)	Age (c)	Intervention (e/c)	Treatment cycle	Stimulation site	Parameters (e1/e2)	Therapeutic instrument models	Country of origin and manufacturer of the instrument	Outcome indicators
Gong^[9]^ 2021	40/40	67.0 ± 2.5	66.5 ± 2.6	B + E/E	4 wk	M1	80%RMT	YRD CCY-1	Yi Ruide Company, Wuhan, China	*
Zhe Zhou^[10]^ 2020	30/28	59.7 ± 10.4	61.8 ± 11.4	C + E/E	3 wk	M1	80%RMT	Rapid2	MAGS-TIM, UK	*,†,‡
Liu^[11]^ 2018	50/50	55.0 ± 12.4	55.6 ± 10.3	B + E/E	2 wk	M1	90%RMT	not mentioned	Yi Ruide Company, Wuhan, China	†
Lian^[12]^ 2023	15/15	61.6 ± 13.7	60.0 ± 11.3	D + E/E	4 wk	M1	70%RMT	YRD CCY-1	Yi Ruide Company, Wuhan, China	*,‡
Zhuang^[13]^ 2022	18/18	62.6 ± 7.7	61.8 ± 10.3	D + E/E	2 wk	M1	80%RMT	MagProX100	MagVenture Corp, Denmark	†,‡
Jing Zhou^[14]^ 2022	12/11	48.8 ± 7.6	55.6 ± 12.6	D + E/E	2 wk	M1	80%AMT	YRD CCY-1	Yi Ruide Company, Wuhan, China	‡
Jiang^[15]^ 2018	13/11/13	61.3 ± 11.3/59.3 ± 9.9	51.8 ± 11.6	D + E/B + E/E	10 d	M1	70%RMT/90%RMT	not mentioned	CR Technology Company, South Korea	*,‡
Zheng^[16]^ 2016	58/54	65.4 ± 13.5	66.2 ± 13.1	B + E/E	4 wk	M1	90%RMT	YRD CCY-1	Yi Ruide Company, Wuhan, China	†
Huang^[17]^ 2023	18/16/18	53.4 ± 11.7/56.4 ± 10.6	52.9 ± 16.2	A + E/C + E/E	2 wk	M1	90%RMT	YRD CCY-1	Yi Ruide Company, Wuhan, China	*,‡
Sun^[18]^ 2023	20/20	57.7 ± 10.5	56.1 ± 11.9	B + E/E	4 wk	M1	90%RMT	YRD CCY-1	Yi Ruide Company, Wuhan, China	*,†,‡
Xiao^[19]^ 2018	15/15	59.1 ± 9.3	60.9 ± 10.2	C + E/E	2 wk	M1	90%RMT	YRD CCY-1	Yi Ruide Company, Wuhan, China	‡
Chen^[20]^ 2019	11/11	52.9 ± 11.1	52.6 ± 8.3	D + E/E	2 wk	Motor cortex	80%AMT	A 70-MM standard coil and a magstim rapid2 magnetic stimulator	Magstim Company, Whitland, UK	*
Shim^[21]^ 2023	14/16	67.3 ± 10.8	63.6 ± 16.1	C + E/E	4 wk	Motor cortex	80%RMT	A 70-MM, 8-shaped coil stimulator	Magstim Company, Whitland, UK	*,‡,§
Luk^[22]^ 2022	12/12	67.3 ± 5.8	65.1 ± 3.1	B + E/E	2 week	M1	90%RMT	A fgure-of-eight coil and a magstim rapid Stimulator	Magstim Company, Whitland, UK	*,§
Yang^[23]^ 2022	12/13	64.0 ± 8.0	64.0 ± 8.0	C + E/E	2 week	M1	100%RMT	YRD CCY-1	Yi Ruide Company, Wuhan, China	*,‡
Haghighi 2021^[24]^	10/10	50.5 ± 9.5	53.9 ± 13.1	C + E/E	3 week	M1	90%RMT	SM9000	Neurosoft, Russia	* §
Kim^[25]^ 2020	40/37	61.2 ± 11.2	62.9 ± 13.1	B + E/E	2 week	M1	100%RMT	An 88-MM-diameter figure-of-eight coil powered by ALTMS	Remed, Korea	*,‡,§
Tretriluxana^[26]^ 2018	8/8	54.3 ± 9.1	60.1 ± 11.6	B + E/E	2 week	M1	90%RMT	A figure-8 air-cooled coil with a magstim rapid2 stimulator	Magstim Company, Whitland, UK	§
Fernández 2016^[27]^	4/3/5	61.4 ± 4.2/58.3 ± 3.2	55.8 ± 10.4	C + E/B + E/E	20 day	Motor cortex	90%AMT/100%AMT	MC-B70	MagPro® magnetic stimulation equipment (Dantec)	‡
Jitka^[28]^ 2015	20/20	65.7 ± 9.9	68.3 ± 10.8	B + E/E	15 day	M1	100% RMT	A 70-MM figure-of-eight coil connected to a magstim super rapid stimulator	Magstim Company, Whitland, UK	†
Sasaki^[29]^ 2014	27/31	66.6 ± 9.5	62.7 ± 10.9	A + E/C + E	5 day	Motor cortex	90%RMT	A 70-MM figure-of-eight coil and a magstim rapid stimulator	Magstim Company, Dyfed, UK	§
Chul Kim^[30]^ 2014	20/20	62.0 ± 12.5	65.1 ± 15.0	B + E/C + E	2 week	M1	90%RMT	MCF-B70	MagVenture, Farum, Denmark	*,‡,§
Seniów^[31]^ 2012	16/17	63.5 ± 8.9	63.4 ± 9.2	B + E/E	3 week	M1	90%RMT	An air-cooled figure-of-eight coil and a magstim rapid stimulator	Magstim Company, Whitland, UK	† *
Khedr^[32]^ 2009	12/12/12	54.7 ± 9.7/ 59.0 ± 13.5	60.0 ± 9.5	B + E/C + E/E	5 day	Motor cortex	100%RMT/130%RMT	Mag-Lite r25	Dantec Medical, Skovelund, Denmark	‡
Chervyakov^[33]^ 2015	11/13/8/10	54.3 ± 11.1/58.7 ± 10.4/60.8 ± 9.6	61.6 ± 11.4	B + E/C + E/A + E/E	2 week	Motor cortex	100%RMT/80%RMT	NBS eXimia Nexstim system	Nexstim Ltd, Helsinki Finland	*,‡
Li^[34]^ 2018	42/43/42	57.9 ± 12.9/54.0 ± 13.4	53.1 ± 13.7	B + E/C + E/E	2 week	M1	80%AMT	YRD CCY-1	Yi Ruide Company, Wuhan, China	† *
Higgins^[35]^ 2013	4/5	74.0 ± 8.0	60.0 ± 11.0	B + E/E	4 week	Motor cortex	110%AMT	Magstim rapid2 stimulator with an air-cooled fgure-8 coil	Rogue Research, Canada	†,§
Wang^[36]^ 2020	15/15/15	60.5 ± 14.1/58.6 ± 10.6	60.5 ± 12.1	B + E/C + E/E	2 week	M1	100%RMT	OSF-4	AOSAIFU, Wuhan, China	*,‡
Kuzu^[37]^ 2021	20/20	56.3 ± 11.5/61.3 ± 9.8	65.0 ± 4.6	B + E/D + E/E	4 week	M1	90%RMT	An 8-shaped 70 MM coil with a magstim rapid2 magnetic stimulator	Magstim, Whitland, Dyfed, UK	*

A = low frequency combined with high frequency rTMS (LF-HF rTMS), AMT = active motor threshold, B = low frequency rTMS (LF-rTMS), c = control group, C = high frequency rTMS (HF-rTMS), D = theta burst stimulation (TBS), e1 = experimental group 1, e2 = experimental group 2, E = routine training, M1 = located in motor cortex area, RMT = resting motor threshold.

* Fugl-Meyer assessment of upper extremity (FMA-UE).

† Wolf motor function test (WMFT).

‡ Modified Barthel Index (MBI).

§ Grip strength; e: experimental group.

### 3.3. Network evidence diagram

**Figure 3.**
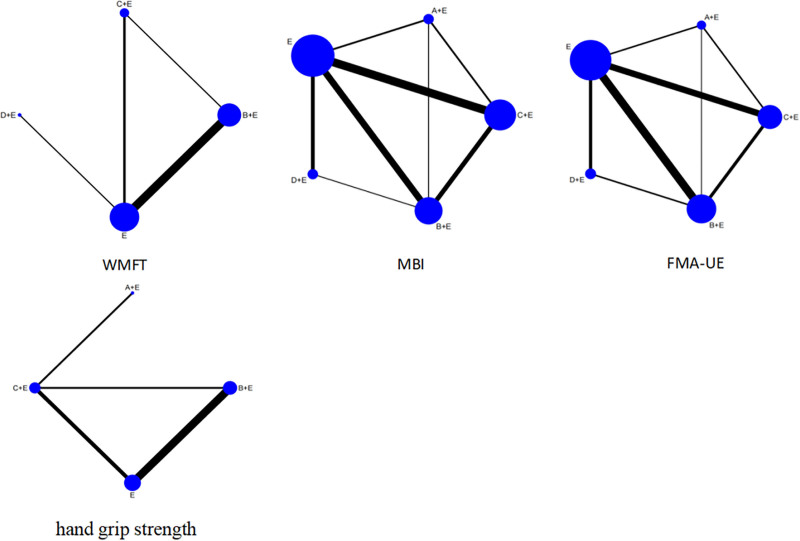
Network evidence map. A: LF-HF rTMS; B: LF-rTMS; C: HF-rTMS; D: TBS; E: routine limb function training. HF-rTMS = high frequency rTMS, LF-HF rTMS = low frequency combined with high frequency rTMS, LF-rTMS = low frequency rTMS, rTMS = repeated transcranial magnetic stimulation, TBS = theta burst stimulation.

The network evidence plots for the 4 outcome indicators are shown in Figure 3, where the size of the dots indicates the number of articles included, and the presence of a straight line between 2 dots demonstrates that there is a direct comparison between the 2 interventions.

### 3.4. Inconsistency test

The global inconsistency test: the indicator WMFT involves a total of 4 interventions, and the inconsistency test yields *P* = .5382; the indicator MBI involves a total of 5 interventions, and the inconsistency test yields *P* = .7438; the grip strength indicator involves a total of 4 interventions, and the inconsistency test yields *P* = .9028; and finally, the indicator FMA-UE involves a total of 5 interventions, and the inconsistency test yields *P* = .5914. From the test results, it can be seen that all 4 indicators have *P* > .05, there is no evidence of inconsistency in the network model, so it can be analyzed using the consistency model. Local inconsistency test: using the node splitting method, it is obtained that all *P* values are > 0.05, therefore, the study does not have obvious local inconsistency. Ring inconsistency test: the 95% CIs of the inconsistency factors all contain 0, *P* > .05, indicating that there is a better consistency exists between direct and indirect evidence.

### 3.5. Results of the meta-analysis

**Figure 4.**
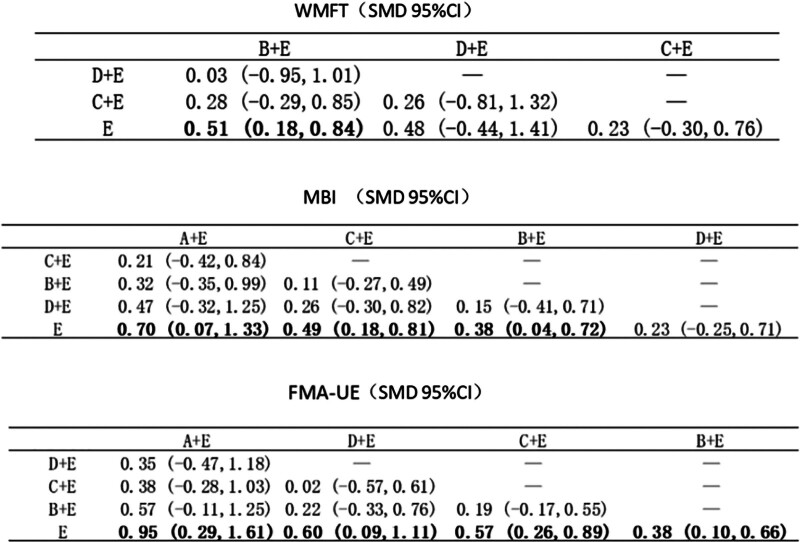
Results of the meta-analysis. A: LF-HF rTMS; B: LF-rTMS; C: HF-rTMS; D: TBS; E: routine limb function training. HF-rTMS = high frequency rTMS, LF-HF rTMS = low frequency combined with high frequency rTMS, LF-rTMS = low frequency rTMS, rTMS = repeated transcranial magnetic stimulation, TBS = theta burst stimulation.

WMFT involved a total of 4 groups, LF-rTMS group, HF-rTMS group, TBS group and control group, of which only the LF-rTMS group was significantly better than the control group (*P* < .05). MBI involved all 5 interventions, and the rest of the groups except the TBS group were significantly better than the control group (*P* < .05). Of the all 5 interventions involved in FMA-UE, all the intervention groups were significantly better than the control group (*P* < .05). Finally, the LF-HF rTMS, LF-rTMS and HF-rTMS groups involved in the grip strength test had nonsignificant but superior differences compared to the control group. The results of the WMFT, MBI and FMA-UE are plotted in Figure 4.

### 3.6. Cumulative ranking probability results

**Figure 5.**
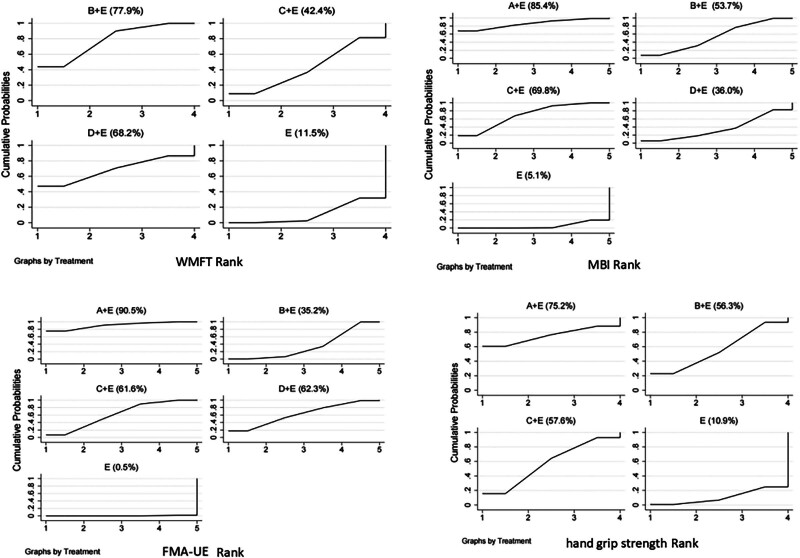
Cumulative probability ranking chart. A: LF-HF rTMS; B: LF-rTMS; C: HF-rTMS; D: TBS; E: routine limb function training. HF-rTMS = high frequency rTMS, LF-HF rTMS = low frequency combined with high frequency rTMS, LF-rTMS = low frequency rTMS, rTMS = repeated transcranial magnetic stimulation, TBS = theta burst stimulation.

In the WMFT, MBI, FMA-UE and grip strength scores, the scores of the 4 outcome indicators were higher, so the higher cumulative area under the curve (SUCRA) value of the intervention indicated a better improvement effect. The SUCRA sorting results of the WMFT scores showed that the LF-rTMS group (77.9%) > TBS group (68.2%) > HF-rTMS group (42.4%) > control group (11.5%). MBI sorting results showed that LF-HF rTMS (85.4%) > HF-rTMS (69.8%) > LF-rTMS (53.7%) > TBS group (36.0%) > control group (5.1%). Both LF-HF rTMS ranked highest in the ranking shown by FMA-UE and grip strength test. The cumulative probability ranking graph is shown in Figure 5.

### 3.7. Publication bias

**Figure 6.**
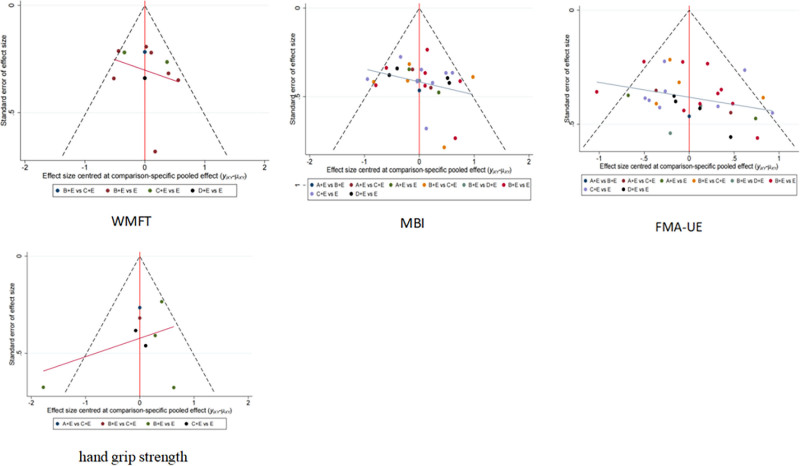
Funnel plot. A: LF-HF rTMS; B: LF-rTMS; C: HF-rTMS; D: TBS; E: routine limb function training. HF-rTMS = high frequency rTMS, LF-HF rTMS = low frequency combined with high frequency rTMS, LF-rTMS = low frequency rTMS, rTMS = repeated transcranial magnetic stimulation, TBS = theta burst stimulation.

The results of the funnel plots of the 4 outcome indicators showed some dots on their outer and bottom sides, suggesting that there may be some small sample size effect and publication bias in this study. The funnel plots of the WMFT, MBI, FMA-UE, and grip strength tests are shown in Figure 6.

## 4. Discussion

As a noninvasive stimulation technique, rTMS is safe, painless and convenient. rTMS can effectively stimulate the central nervous system and produce a wide range of changes through interactions with neurons, including changes in membrane potential and changes in signal conduction. rTMS has the effect of promoting the restoration of interhemispheric equilibrium and the functional reorganization of the brain, which is important for the improvement of limb function. In recent years, many clinical controlled trials and meta-analyses have demonstrated the effectiveness of rTMS in the treatment of upper limb dysfunction after stroke, but there are fewer comparative analyses of the effectiveness of hand function therapy alone. In terms of stimulation protocols, more comprehensive comparisons between different rTMS stimulation protocols are lacking, and there is a lack of comparisons between 2 magnetic stimulation means combined such as LF-HF rTMS versus other protocols. A total of 29 papers were included in this study to systematically evaluate 4 intervention protocols for rTMS.

The analysis found that the rTMS interventions or some of the interventions in the article achieved more significant results in terms of the scores of WMFT, MBI, and FMA-UE, which is consistent with the conclusion of a recent review^[38]^ that found that rTMS had better results in treating the upper limb and hand function after stroke. This may be related to the theory mentioned in the interhemispheric competition model,^[39–41]^ rTMS is able to improve limb movement in patients by promoting cortical excitability on the affected side or inhibiting cortical excitability on the healthy side.^[42]^

Hand function is mainly based on fine motor movements, and its area involved in the cerebral cortex is twice as large as that of the lower limbs, and the complexity of the function determines the difficulty of the hand function rehabilitation process.^[43,44]^ The present study also found relatively few literatures or methods to study hand function recovery, which indirectly indicates the difficulty of hand rehabilitation and the small number of researchers. In contrast, the current study found a significant efficacy of LF-rTMS combined with functional training after analyzing the included literature, which is consistent with Tretriluxana et al^[45]^ who found that LF-rTMS has a better effect on improving hand function. This efficacy may be related to the fact that LF-rTMS acts on the primary motor cortex (M1) region of the healthy brain to continuously inhibit the hyperexcitability of the healthy hemisphere, and that repeated rehabilitation training of the hand continually reinforces the feedback and thus stimulates the synapses of the neurons on the affected side to form new connections.

Currently, it is rare for the comparison of the stimulation combining 2 different rTMS with other single rTMS stimulation, while in the present study, we found that the LF-HF rTMS stimulation protocol was optimal for the sequencing of MBI, FMA-UE, and grip strength, and achieved significant therapeutic effects compared with the control group in terms of MBI and FMA-UE (*P* < .05), which may be related to the fact that LF-rTMS inhibited the excitability of the healthy side of the brain while HF-rTMS increased the excitability of the affected side of the brain. And the study of Long et al^[6]^ also pointed out that the regimen of high frequency on the affected side combined with low frequency rTMS on the healthy side had a more pronounced effect on the improvement of motor function than a single low frequency, which suggests that LF-HF rTMS can be used as an important reference regimen for the treatment of poststroke limb dysfunction.

Although transcranial magnetism has certain therapeutic effects, there are some potential adverse reactions to its application, such as causing dizziness, so the patient’s condition should be closely observed during the treatment process, and corresponding countermeasures should be taken in a timely manner when the adverse reactions occur.

This meta-analysis has the following limitations: It mainly included Chinese and English literature, and there may be a risk of incomplete search; positive results are more likely to be published, so there may be publication bias; some interventions included relatively few articles, such as the TBS program. Some of the included studies did not state the specific blinding method, and the results may have some bias.

## 5. Conclusion

In summary, rTMS was found to have a positive effect on the improvement of limb function and daily living ability after stroke, of which LF-rTMS has a greater potential for the improvement of hand function, while the LF-HF rTMS protocol with the combination of 2 rTMS stimulation modalities achieved a relatively better sequencing in terms of the improvement of FMA-UE, MBI and grip strength, respectively. In addition, further exploration of rTMS protocols requires larger sample sizes and more treatment options for future research.

Despite the more positive conclusions of our study, there is continuing uncertainty regarding the clinical implementation of these treatments, and further robust studies will be needed in the future to validate the long-term efficacy of the respective regimens.

## Author contributions

**Conceptualization:** Yue Shen, Jinchao Du, Xiaoduo Yao.

**Data curation:** Yue Shen, Jinchao Du.

**Formal analysis:** Yue Shen, Xiaoduo Yao, Jiqin Tang.

**Investigation:** Jinchao Du, Xiaoduo Yao.

**Methodology:** Yue Shen, Xiaoduo Yao.

**Project administration:** Jiqin Tang.

**Software:** Yue Shen.

**Supervision:** Jinchao Du, Jiqin Tang.

**Writing – original draft:** Yue Shen.

**Writing – review & editing:** Yue Shen, Jiqin Tang.
